# Potential influence of long non-coding RNA *NEAT1* genetic variants and expression levels on the progression of prostate cancer

**DOI:** 10.7150/jca.137994

**Published:** 2026-08-10

**Authors:** Cheng-En Mei, Chia-Yen Lin, Shian-Shiang Wang, Shih-Chi Su, Heng-Hsiung Wu, Lun-Ching Chang, Chih-Hsin Tang, Shun-Fa Yang

**Affiliations:** 1 Institute of Medicine, Chung Shan Medical University, Taichung, Taiwan; 2 Department of Urology, Taichung Veterans General Hospital, Taichung, Taiwan; 3 School of Medicine, Chung Shan Medical University, Taichung, Taiwan; 4 Whole-Genome Research Core Laboratory of Human Diseases, Chang Gung Memorial Hospital, Keelung, Taiwan; 5 Department of Medical Biotechnology and Laboratory Science, College of Medicine, Chang Gung University, Taoyuan, Taiwan; 6 Program for Cancer Biology and Drug Discovery, China Medical University, Taichung, Taiwan; 7 Department of Mathematics and Statistics, Florida Atlantic University, Florida, USA; 8 Department of Pharmacology, School of Medicine, China Medical University, Taichung, Taiwan; 9 Department of Medical Laboratory Science and Biotechnology, Asia University, Taichung, Taiwan; 10 Chinese Medicine Research Center, China Medical University, Taichung, Taiwan; 11 Department of Medical Research, Chung Shan Medical University Hospital, Taichung, Taiwan

**Keywords:** NEAT1, long non-coding RNA (lncRNA), genetic polymorphism, perineural invasion, PSA.

## Abstract

Prostate cancer is a leading malignancy in men worldwide, with disease aggressiveness varying widely among patients. Long non-coding RNA NEAT1 has emerged as a key regulator of tumor progression, yet the impact of its genetic variants on prostate cancer susceptibility and aggressiveness remains unclear. In this study, we investigated the association of *NEAT1* polymorphisms with clinicopathological features in 693 prostate cancer patients. Patients were stratified by PSA levels (≤ 10 ng/mL vs. > 10 ng/mL), and genotypes of *NEAT1* rs3741384, rs512715, and rs3825071 were determined. Among the analyzed *NEAT1* SNPs, rs3825071 was significantly associated with PSA levels and perineural invasion in patients with prostate cancer. In the high-PSA subgroup, carriers of the CT and TT genotypes exhibited a lower likelihood of elevated PSA (> 10 ng/mL) (AOR = 0.794, p = 0.011) and a reduced risk of perineural invasion (OR = 0.470, p = 0.007). Functional analyses using the GTEx and TCGA datasets revealed genotype-dependent alterations in NEAT1 expression, with significant upregulation observed in prostate cancer tissues, particularly in cases with higher Gleason scores and lymph node metastasis. Collectively, our results suggest that *NEAT1* rs3825071 T alleles confer a protective effect and highlight its potential as a genetic biomarker for prostate cancer progression.

## 1. Introduction

Prostate cancer is one of the most common malignant tumors in men worldwide and is a major cause of cancer-related mortality. In the United States alone, approximately 313,780 new cases and 35,770 deaths were reported in 2025, highlighting its substantial global health burden 1. The risk of prostate cancer increases significantly with age, particularly after 55 years, and marked differences exist among ethnic groups. Although most prostate cancer patients present with localized disease at diagnosis, survival outcomes differ dramatically once the disease becomes metastatic 2, 3.

Serum prostate-specific antigen (PSA) testing remains a central tool for prostate cancer screening and early diagnosis. Its widespread use has significantly increased detection rates and led to more than one million prostate biopsy procedures annually^4^. However, PSA has limited specificity in distinguishing clinically significant cancer from indolent lesions, especially in patients with PSA levels below 10 ng/mL, in whom the cancer detection rate is only 22% to 27% 5, 6. This limitation leads to overdiagnosis, overtreatment, and unnecessary invasive procedures, while still failing to reliably predict tumor aggressiveness or metastatic potential^7^-^9^. Therefore, it is important to improve the clinical interpretation of PSA and to integrate additional prognostic indicators to enhance risk stratification and optimize the management of prostate cancer.

Long non-coding RNAs (lncRNAs) are transcripts longer than 200 nucleotides that lack protein-coding capacity. In recent years, they have been recognized as key regulators of gene expression, cell cycle progression, differentiation, and tumorigenesis 10-13. Among these, NEAT1 (nuclear enriched abundant transcript 1) has attracted considerable attention due to its high nuclear abundance and its essential role as a structural scaffold for the formation of paraspeckle nuclear subdomains 14. Emerging evidence indicates that NEAT1 promotes tumor progression through multiple mechanisms, including miRNA sponging, modulation of signaling pathways, regulation of epithelial-mesenchymal transition (EMT), maintenance of cancer stemness, metabolic reprogramming, and autophagy regulation 15, 16.

NEAT1 is frequently dysregulated across a variety of malignancies, including prostate cancer 14, 17-20. Recent studies have demonstrated that NEAT1 plays a critical role in prostate cancer, where its dysregulated expression is closely associated with enhanced tumor proliferation, invasion, metastasis, and poor clinical outcomes 21, 22. For example, NEAT1 is upregulated in prostate cancer and promotes bone metastasis through an N6-methyladenosine (m6A)-dependent mechanism 21. Furthermore, Xu et al. reported that NEAT1 promotes prostate cancer progression by regulating the miR-582-5p/EZH2 signaling axis 22. In addition, several single nucleotide polymorphisms (SNPs) in *NEAT1*, including rs3825071, have been associated with clinicopathological characteristics of tongue cancer and colorectal cancer progression 23, 24. Moreover, accumulating evidence suggests that *NEAT1* polymorphisms, including rs3825071 and rs7943779, may also contribute to gastric cancer susceptibility 25, underscoring their potential clinical significance. Although the functional roles of NEAT1 in prostate cancer have begun to emerge, the impact of its genetic variants on prostate cancer susceptibility has yet to be elucidated. In this study, we performed a hypothesis-driven investigation to examine the association between *NEAT1* SNPs and prostate cancer tumorigenesis.

## 2. Materials and Methods

### 2.1 Subjects

This retrospective study included 693 patients with prostate cancer who underwent robot-assisted radical prostatectomy with bilateral standard pelvic lymph node dissection at Taichung Veterans General Hospital. The study was approved by the Institutional Review Board (IRB No. CE19062A), and written informed consent was obtained from all participants. Clinical and pathological data, including age at diagnosis, initial PSA level, Gleason score/grade group, TNM stage (AJCC 8th edition), D'Amico risk classification, and pathological features (seminal vesicle, perineural, and lymphovascular invasion), were collected from medical records 26. Patients were stratified into two groups based on initial PSA level: 330 patients with PSA ≤ 10 ng/mL (PSA ≤ 10 group) and 363 patients with PSA > 10 ng/mL (PSA > 10 group). The cutoff value of 10 ng/mL was selected because it is a clinically established threshold widely used for prostate cancer risk stratification in current clinical practice guidelines.

### 2.2 Specimen collection and genomic DNA extraction

Genomic DNA was extracted using the QIAamp DNA Blood Mini Kit (Qiagen, Valencia, CA, USA) according to the manufacturer's instructions and stored at -80 °C. DNA quality and concentration were assessed using a Nanodrop-2000 spectrophotometer (Thermo Scientific, Waltham, MA, USA), and high-quality DNA was used as a template for downstream analyses according to previously described 27.

### 2.3 Selection and Analysis of Neat1 Genetic Polymorphisms

In this study, three *NEAT1* SNPs (rs3741384, rs512715, and rs3825071) were selected based on data from the International HapMap Project database and previous studies demonstrating their potential biological significance and associations with cancer susceptibility and progression in various malignancies 23-25. Genetic polymorphisms of *NEAT1* (rs3741384, rs512715, and rs3825071) were genotyped using TaqMan SNP Genotyping Assays (Applied Biosystems) on an ABI StepOnePlus Real-Time PCR System 24. PCR reactions were performed following standard protocols, and genotypes were analyzed using SDS software.

### 2.4 Statistical analysis

Continuous and categorical variables were compared using Student's t-test and chi-square or Fisher's exact test, respectively. Associations between genotypes and disease risk or clinicopathological features were evaluated using logistic regression models, with results presented as odds ratios (ORs) or adjusted ORs (AORs) with 95% confidence intervals (CIs), adjusting for age and gender. Statistical analyses were performed using SAS software (version 9.1; SAS Institute, Cary, NC, USA), with p < 0.05 considered statistically significant.

## 3. Results

### 3.1 Characteristics of the study participants

A total of 693 patients with prostate cancer were enrolled, including 330 with PSA levels < 10 ng/mL and 363 with PSA levels > 10 ng/mL (Table 1). Patients with PSA > 10 ng/mL were more likely to be older than 65 years (62.8% vs. 51.8%, p = 0.003) and exhibited more aggressive disease characteristics, including higher pathologic Gleason grade group (52.3% vs. 26.4%), advanced clinical T stage (20.4% vs. 6.1%), and increased lymph node involvement at both clinical and pathological levels. In addition, the high PSA group showed significantly higher rates of advanced pathological T stage (61.2% vs. 31.2%), seminal vesicle invasion (32.0% vs. 9.4%), perineural invasion (80.2% vs. 65.8%), lymphovascular invasion (22.9% vs. 8.2%), high-risk D'Amico classification (70.5% vs. 28.2%), and biochemical recurrence (41.6% vs. 20.3%) (all p < 0.001).

### 3.2 Association between NEAT1 Polymorphisms and PSA Levels

The distributions of *NEAT1* genotypes among patients stratified by PSA levels are shown in Table 2. No significant differences were observed in the genotype frequencies of *NEAT1* rs3741384 and rs512715 between the two PSA groups. After adjustment for clinicopathological variables, neither rs3741384 nor rs512715 polymorphisms were significantly associated with PSA levels. In contrast, a significant association was observed for *NEAT1* rs3825071. Patients carrying the CT genotype had a significantly lower likelihood of having PSA levels >10 ng/mL compared to those with the CC genotype (AOR = 0.633, 95% CI: 0.438-0.915, p = 0.015). Similarly, individuals with combined CT+TT genotypes also showed a reduced risk (AOR = 0.794, 95% CI: 0.664-0.948, p = 0.011) (Table 2).

### 3.3 Association between NEAT1 rs3825071 Polymorphism and Clinicopathological Characteristics

The associations between *NEAT1* rs3825071 polymorphism and clinicopathological characteristics are presented in Table 3. A significant association was observed for perineural invasion. Patients carrying *NEAT1* rs3825071 CT+TT genotypes had a significantly lower risk of perineural invasion compared to those with the CC genotype (OR = 0.683, 95% CI: 0.478-0.976, p = 0.036). In contrast, most clinical parameters, including pathologic Gleason grade group, clinical and pathologic T stage, pathologic N stage, seminal vesicle invasion, lymphovascular invasion, D'Amico classification, and biochemical recurrence, were not significantly associated with genotypes.

Stratified analyses according to PSA levels are shown in Table 4. Among patients with PSA levels < 10 ng/mL, no significant associations were observed between rs3825071 genotypes and clinicopathological characteristics. In contrast, among patients with PSA levels > 10 ng/mL, the rs3825071 polymorphism was significantly associated with perineural invasion. Patients with CT+TT genotypes had a significantly lower risk of perineural invasion compared to those with the CC genotype (OR = 0.470, 95% CI: 0.271-0.816, p = 0.007). No significant associations were found between rs3825071 polymorphism and other clinicopathological features in either PSA subgroup.

### 3.4 Functional and clinical relevance of NEAT1 rs3825071 in prostate cancer

**Figure 1:**
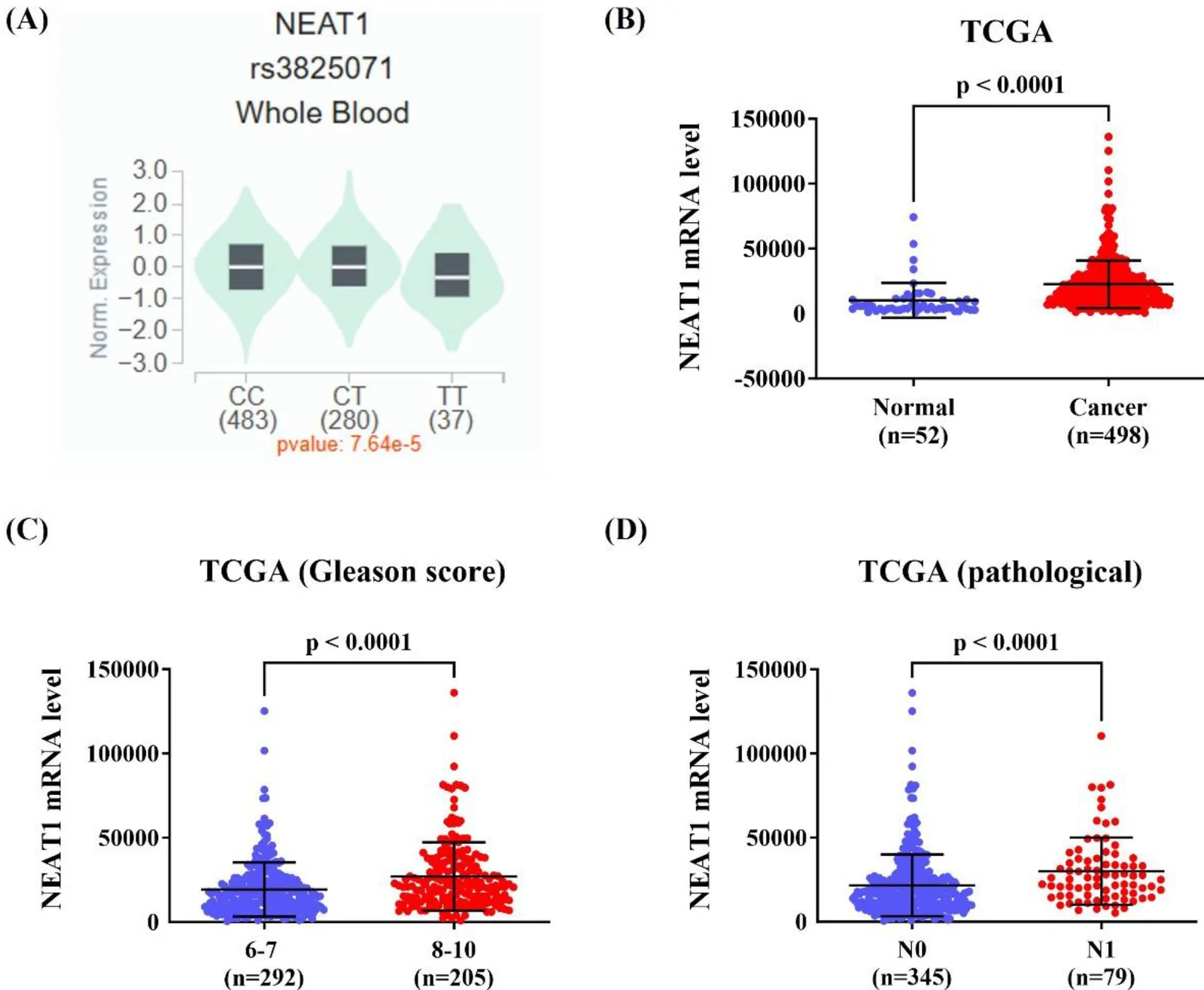
** Functional impact of *NEAT1* rs3825071 in prostate cancer. (A)** NEAT1 expression in whole blood stratified by rs3825071 genotype in the GTEx database (p = 7.64 × 10⁻⁵). **(B)** NEAT1 expression in prostate cancer versus normal tissue from TCGA. **(C)** Comparison of NEAT1 expression across Gleason score groups in prostate cancer. **(D)** Comparison of NEAT1 expression based on lymph node metastasis status in prostate cancer.

Following the identification of an association between *NEAT1* rs3825071 and perineural invasion in prostate cancer, we further explored the potential functional relevance of NEAT1 using publicly available transcriptomic data from the Genotype-Tissue Expression (GTEx) and The Cancer Genome Atlas (TCGA) databases. In the GTEx database, NEAT1 expression in whole blood varied significantly across rs3825071 genotypes (p = 7.64 × 10⁻⁵) (Figure 1A). Analysis of prostate cancer data from TCGA showed NEAT1 upregulation in prostate cancer relative to normal tissue (p < 0.001) (Figure 1B), with higher expression associated with higher Gleason scores (p < 0.001) (Figure 1C) and lymph node metastasis (p < 0.001) (Figure 1D). These results reinforce the observed genetic associations and suggest that *NEAT1* polymorphisms may drive prostate cancer progression through modulation of gene expression.

## 4. Discussion

In the present study, we demonstrated that elevated PSA levels at diagnosis were significantly associated with adverse clinicopathological features in patients with prostate cancer. Individuals with PSA >10 ng/mL exhibited higher Gleason grade, more advanced tumor stage, increased lymph node involvement, and higher rates of seminal vesicle invasion, perineural invasion, lymphovascular invasion, and biochemical recurrence. With respect to genetic factors, we found that *Neat1* rs3825071 was significantly associated with PSA stratification, with CT and CT+TT genotypes showing a reduced likelihood of presenting with PSA >10 ng/mL. This finding suggests a potential protective role of this polymorphism in modulating prostate cancer progression.

NEAT1 expression is frequently dysregulated in prostate cancer 21, 22. In addition to aberrant NEAT1 expression, NEAT1 polymorphisms have been associated with clinical outcomes in various cancers 23, 24, 28. Our findings indicate that *NEAT1* rs3825071 may influence NEAT1 expression through both regulatory and functional mechanisms. Consistent with our findings in the GTEx database (Figure 1A), Gerile et al. reported that NEAT1 expression in individuals with the CT and TT genotypes was significantly lower than in those with the CC genotype of rs3825071 in the HIV-1 group 29. These results highlight rs3825071 as a potential expression quantitative trait locus (eQTL).

NEAT1 participates in a wide range of cancer-related biological activities, such as gene transcription control, chromatin organization, and cellular responses to stress in prostate cancer^30^, ^31^. In our analysis, individuals with the Neat1 rs3825071 CT or TT genotypes showed a significantly reduced likelihood of perineural invasion compared with those carrying the CC genotype. Previous studies have reported expression patterns of NEAT1 in prostate cancer 20, 31-33. For example, Chakravarty et al., reported that NEAT1 as a prognostic biomarker for aggressive prostate cancers^32^. Moreover, Bai et al. found that NEAT1 was significantly upregulated in prostate cancer tissues and correlated with TNM stage, lymph node and distant metastasis, and Gleason score 33. In our analysis of human prostate cancer using data from TCGA, NEAT1 expression was elevated in tumor tissues compared with normal counterparts and was further increased in cases with lymph node metastasis. These findings suggest that the *NEAT1* rs3825071 polymorphism may contribute to perineural invasion in prostate cancer, potentially through modulation of NEAT1 expression. Collectively, these observations support the notion that genetic variation in *NEAT1* can influence its biological function and, in turn, affect tumor aggressiveness, particularly with respect to neural invasion. Nevertheless, further studies are warranted to validate these findings, including analyses with larger sample sizes, longer follow-up durations, and independent cohort validation, especially in Asian populations and publicly available datasets.

In conclusion, our findings indicate that the *NEAT1* rs3825071 polymorphism is associated with PSA levels and perineural invasion in patients with prostate cancer. The *NEAT1* rs3825071 CT and TT genotypes appear to confer a protective effect, particularly in patients with elevated PSA levels. These results suggest that *NEAT1* rs3825071 may serve as a potential genetic biomarker for disease aggressiveness. Further large-scale and functional studies are warranted to elucidate the underlying mechanisms and to validate its clinical utility.

## Figures and Tables

**Table 1:** The distributions of demographical characteristics in 693 patients with prostate cancer.

Variable	PSA at diagnosis (ng/ml)			
	< 10 (n = 330)	> 10 (n = 363)		p value
Age at diagnosis (years				
< 65	159 (48.2 %)		135 (37.2 %)	P = 0.003*
> 65	171 (51.8 %)		228 (62.8 %)	
Pathologic Gleason grade group				
1+2	243 (73.6 %)		173 (47.7 %)	P < 0.001*
3+4+5	87 (26.4 %)		190 (52.3 %)	
Clinical T stage				
1+2	310 (93.9 %)		289 (79.6 %)	P < 0.001*
3+4	20 (6.1 %)		74 (20.4 %)	
Clinical N stage				
N0	327 (99.1 %)		352 (97.0 %)	P = 0.047*
N1	3 (0.9 %)		11 (3.0 %)	
Pathologic T stage				
2	227 (68.8 %)		141 (38.8 %)	P < 0.001*
3+4	103 (31.2 %)		222 (61.2 %)	
Pathologic N stage				
N0	315 (95.5 %)		318 (87.6 %)	P < 0.001*
N1	15 (4.5 %)		45 (12.4 %)	
Seminal vesicle invasion				
No	299 (90.6 %)		247 (68.0 %)	P < 0.001*
Yes	31 (9.4 %)		116 (32.0 %)	
Perineural invasion				
No	113 (34.2 %)		72 (19.8%)	P < 0.001*
Yes	217 (65.8 %)		291 (80.2 %)	
Lymphovascular invasion				
No	303 (91.8 %)		280 (77.1 %)	P < 0.001*
Yes	27 (8.2 %)		83 (22.9 %)	
D'Amico classification				
Low risk/ Intermediate risk	237 (71.8 %)		107 (29.5 %)	P < 0.001*
High risk	93 (28.2 %)		256 (70.5 %)	
Biochemical recurrence				
No	263 (79.7 %)		212 (58.4 %)	P < 0.001*
Yes	67 (20.3 %)		151 (41.6 %)	
				

* p value < 0.05 as statistically significant.

**Table 2:** Distribution frequency of *NEAT1* genotypes in 693 patients with prostate cancer.

Variable	PSA at diagnosis (ng/ml)			
	< 10 (n = 330)	> 10 (n = 363)	AOR (95% CI)	p value
rs3741384				
GG	223 (67.6%)	255 (70.2%)	1.000 (reference)	
GA	94 (28.5%)	101 (27.8%)	0.918 (0.638-1.319)	P = 0.642
AA	13 (3.9%)	7 (2.0%)	0.489 (0.177-1.357)	P = 0.170
GA+AA	107 (32.4%)	108 (29.8%)	0.931 (0.781-1.110)	P = 0.427
rs512715				
GG	169 (51.2%)	188 (51.8%)	1.000 (reference)	
GC	133 (40.3%)	143 (39.4%)	0.930 (0.661-1.308)	P = 0.677
CC	28 (8.5%)	32 (8.8%)	1.100 (0.608-1.988)	P = 0.753
GC+CC	161 (48.8%)	175 (48.2%)	0.979 (0.833-1.151)	P = 0.796
rs3825071				
CC	212 (64.2%)	272 (74.9%)	1.000 (reference)	
CT	107 (32.4%)	83 (22.9%)	0.633 (0.438-0.915)	P = 0.015*
TT	11 (3.3%)	8 (2.2%)	0.600 (0.222-1.620)	P = 0.313
CT+TT	118 (35.8%)	91 (25.1%)	0.794 (0.664-0.948)	P = 0.011*

The adjusted odds ratios (AORs) with their 95% confidence intervals (CIs) were estimated by multiple logistic regression models after controlling for pathologic Gleason grade group, clinical T stage, clinical N stage, pathologic T stage, pathologic N stage, seminal vesicle invasion, perineural invasion, lymphovascular invasion, D'Amico classification and biochemical recurrence.

**Table 3:** Odds ratios (ORs) and 95% confidence intervals (CIs) of the clinical status and *NEAT1* rs3825071 genotypic frequencies in 693 patients with prostate cancer.

Variable	rs3825071			
	CC (*N* = 484)	CT + TT (*N* = 209)	OR (95% CI)	p value
Pathologic Gleason grade group				
1+2	284 (58.7%)	132 (63.2%)	1.000	0.269
3+4+5	200 (41.3%)	77 (36.8%)	0.828 (0.593~1.157)	
Clinical T stage				
1+2	414 (85.5%)	185 (88.5%)	1.000	0.293
3+4	70 (14.5%)	24 (11.5%)	0.767 (0.468~1.259)	
Pathologic T stage				
2	249 (51.4%)	119 (56.9%)	1.000	0.184
3+4	235 (46.6%)	90 (43.1%)	0.801 (0.578~1.111)	
Pathologic N stage				
N0	447 (92.4%)	186 (89.0%)	1.000	0.149
N1	37 (7.6%)	23 (11.0%)	1.494 (0.864~2.584)	
Seminal vesicle invasion				
No	374 (77.3%)	172 (82.3%)	1.000	0.138
Yes	110 (22.7%)	37 (17.7%)	0.731 (0.483~1.106)	
Perineural invasion				
No	118 (24.4%)	67 (32.1%)	1.000	0.036*
Yes	366 (75.6%)	142 (67.9%)	0.683 (0.478~0.976)	
Lymphovascular invasion				
No	406 (83.9%)	177 (84.7%)	1.000	0.790
Yes	78 (16.1%)	32 (15.3%)	0.941 (0.601~1.472)	
D'Amico classification				
Low risk/ Intermediate risk	236 (48.8%)	108 (51.7%)	1.000	0.481
High risk	248 (51.2%)	101 (48.3%)	0.890 (0.643~1.231)	
Biochemical recurrence				
No	329 (68.0%)	146 (69.9%)	1.000	0.625
Yes	155 (32.0%)	63 (30.1%)	0.916 (0.644~1.302)	

ORs with their 95% CIs were estimated by logistic regression models. * p < 0.05 as statistically significant.

**Table 4:** Odds ratios (ORs) and 95% confidence intervals (CIs) of the clinical status and *NEAT1* rs3825071 genotypic frequencies in 693 prostate cancer patients with different PSA at diagnosis.

Variable	PSA at diagnosis < 10 ng/mL (n = 330)				PSA at diagnosis >10 ng/mL (N = 363)			
	CC (*N* = 212)	CT + TT (*N* = 118)	OR (95% CI)	p value	CC (*N* = 272)	CT + TT (*N* = 91)	OR (95% CI)	p value
Pathologic Gleason grade group								
1+2	162 (76.4%)	81 (68.6%)	1.000	0.125	122 (44.9%)	51 (56.0%)	1.000	0.064
3+4+5	50 (23.6%)	37 (31.4%)	1.480 (0.896~2.444)		150 (55.1%)	40 (44.0%)	0.638 (0.396~1.029)	
Clinical T stage								
1+2	201 (94.8%)	109 (92.4%)	1.000	0.374	213 (78.3%)	76 (83.5%)	1.000	0.286
3+4	11 (5.2%)	9 (7.6%)	1.509 (0.607~3.753)		59 (21.7%)	15 (16.5%)	0.713 (0.382~1.330)	
Pathologic T stage								
2	150 (70.8%)	77 (65.3%)	1.000	0.301	99 (36.4%)	42 (46.2%)	1.000	0.098
3+4	62 (29.2%)	41 (34.7%)	1.288 (0.797~2.084)		173 (63.6%)	49 (53.8%)	0.668 (0.413~1.079)	
Pathologic N stage								
N0	206 (97.2%)	109 (92.4%)	1.000	0.084	241 (88.6%)	77 (84.6%)	1.000	0.318
N1	6 (2.8%)	9 (7.6%)	2.835 (0.983~8.172)		31 (11.4%)	14 (15.4%)	1.413 (0.715~2.794)	
Seminal vesicle invasion								
No	194 (91.5%)	105 (89.0%)	1.000	0.451	180 (66.2%)	67 (73.6%)	1.000	0.187
Yes	18 (8.5%)	13 (11.0%)	1.334 (0.629~2.830)		92 (33.8%)	24 (26.4%)	0.701 (0.413~1.190)	
Perineural invasion								
No	73 (34.4%)	40 (33.9%)	1.000	0.922	45 (16.5%)	27 (29.7%)	1.000	0.007*
Yes	139 (65.6%)	78 (66.1%)	1.024 (0.637~1.647)		227 (83.5%)	64 (70.3%)	0.470 (0.271~0.816)	
Lymphovascular invasion								
No	197 (92.9%)	106 (89.8%)	1.000	0.326	209 (76.8%)	71 (78.0%)	1.000	0.816
Yes	15 (7.1%)	12 (10.2%)	1.487 (0.671~3.292)		63 (23.2%)	20 (22.0%)	0.934 (0.528~1.653)	
D'Amico classification								
Low risk/ Intermediate risk	158 (74.5%)	79 (66.9%)	1.000	0.142	78 (28.7%)	29 (31.9%)	1.000	0.563
High risk	54 (25.5%)	39 (33.1%)	1.444 (0.883~2.364)		194 (71.3%)	62 (68.1%)	0.860 (0.514~1.436)	
Biochemical recurrence								
No	173 (81.6%)	90 (76.3%)	1.000	0.248	156 (57.4%)	56 (61.5%)	1.000	0.483
Yes	39 (18.4%)	28 (23.7%)	1.380 (0.798~2.388)		116 (42.6%)	35 (38.5%)	0.841 (0.517~1.366)	

ORs with their 95% CIs were estimated by logistic regression models. * p < 0.05 as statistically significant.
